# Multiple adverse outcomes following first discharge from inpatient psychiatric care: a national cohort study

**DOI:** 10.1016/S2215-0366(19)30180-4

**Published:** 2019-07

**Authors:** Florian Walter, Matthew J Carr, Pearl L H Mok, Sussie Antonsen, Carsten B Pedersen, Louis Appleby, Seena Fazel, Jenny Shaw, Roger T Webb

**Affiliations:** a Centre for Mental Health & Safety, Division of Psychology & Mental Health, School of Health Sciences, Faculty of Biology, Medicine, and Health, The University of Manchester and Manchester Academic Health Sciences Centre, Manchester, UK; b National Institute for Health Research Greater Manchester Patient Safety Translational Research Centre, Manchester, UK; c National Centre for Register-based Research and Centre for Integrated Register-based Research, Department of Economics and Business Economics, Aarhus University, Aarhus, Denmark; d Department of Psychiatry, Medical Sciences Division, University of Oxford, Warneford Hospital, Oxford, UK

## Abstract

**Background:**

Discharged psychiatric inpatients are at elevated risk of serious adverse outcomes, but no previous study has comprehensively examined an array of multiple risks in a single cohort.

**Methods:**

We used data from the Danish Civil Registration System to delineate a cohort of all individuals born in Denmark in 1967–2000, who were alive and residing in Denmark on their 15th birthday, and who had been discharged from their first inpatient psychiatric episode at age 15 years or older. Each individual in the discharged cohort was matched on age and sex with 25 comparators without a history of psychiatric admission. Data linked to each individual were also obtained from the Psychiatric Central Research Register, Register of Causes of Death, National Patient Register, and the National Crime Register. We used survival analysis techniques to estimate absolute and relative risks of all-cause mortality, suicide, accidental death, homicide victimisation, homicide perpetration, non-fatal self-harm, violent criminality, and hospitalisation following violence, until Dec 31, 2015.

**Findings:**

We included 62 922 individuals in the discharged cohort, and 1 573 050 matched comparators. Risks for each of all eight outcomes examined were markedly elevated in the discharged cohort relative to the comparators. Within 10 years of first discharge, the cumulative incidence of death, self-harm, committing a violent crime, or hospitalisation due to interpersonal violence was 32·0% (95% CI 31·6–32·5) in the discharged cohort (37·1% [36·5–37·8] in men and 27·2% [26·7–27·8] in women). Absolute risk of at least one adverse outcome occurring within this timeframe were highest in people diagnosed with a psychoactive substance use disorder at first discharge (cumulative incidence 49·4% [48·4–50·4]), and lowest in those diagnosed with a mood disorder (24·4% [23·6–25·2]). For suicide and non-fatal self-harm, risks were especially high during the first 3 months post-discharge, whereas risks for accidental death, violent criminality, and hospitalisation due to violence were more constant throughout the 10-year follow-up.

**Interpretation:**

People discharged from inpatient psychiatric care are at higher risk than the rest of the population for a range of serious fatal and non-fatal adverse outcomes. Improved inter-agency liaison, intensive follow-up immediately after discharge, and longer-term social support are indicated.

**Funding:**

Medical Research Council, European Research Council, and Wellcome Trust.

## Introduction

People discharged from inpatient psychiatric care are more susceptible to experiencing serious adverse outcomes, including all-cause mortality, suicide and non-fatal self-harm, and accidental death.^1^, ^2^, ^3^, ^4^, ^5^ These individuals are also more likely to become victims of^6^, ^7^, ^8^, ^9^, ^10^ and to perpetrate^11^, ^12^, ^13^, ^14^, ^15^ violent crimes, including homicide; however, without accurate quantification of absolute and relative risks, stigma towards people who experience mental illness^16^ cannot be effectively tackled. To date, these adverse events have predominantly been investigated and reported individually as a single outcome from each respective study. Therefore, because of between-cohort differences in study populations and designs, exposure measures, outcome classifications, ascertainment procedures, and other aspects, this body of literature does not enable direct and comprehensive comparison of risks across the range of pertinent outcomes.

We aimed to generate new information concerning risks for the following fatal and non-fatal adverse outcomes among individuals discharged from their first inpatient psychiatric treatment episode: all-cause mortality, suicide, accidental death, homicide victimisation, homicide perpetration, non-fatal self-harm, violent criminality, and hospitalisation for injuries sustained through serious interpersonal violence (hereafter referred to as hospitalisation due to violence). We used Danish national registry data to ensure sufficient statistical power and precision for investigating even homicide victimisation and perpetration—the two rarest outcomes examined.

In contrast to previously published registry investigations, we did not restrict examination of the study cohort to specific mental disorders such as schizophrenia,^15^ but instead investigated all patients discharged from inpatient psychiatric care. Incidence rates and relative risks were estimated separately according to follow-up period (ranging from the first 3 months to 10 years or more post-discharge). An array of adverse outcomes was investigated in a single cohort with comparison of absolute and relative risks across the various endpoints examined—a novel and informative paradigm that is currently absent from the published literature. Such a comparison is informative in capturing the broader range of risks faced by former psychiatric inpatients, to thereby inform service planning and policy. We hypothesised that risks for each adverse outcome examined would be increased in former psychiatric inpatients compared with the risks in individuals of the same age and sex without a history of inpatient psychiatric care. Suicide risk is known to be especially raised soon after discharge, before declining over time,^3^ albeit with risk in the longer term remaining considerably higher than that in the remainder of the population. We predicted that a similar temporal pattern would be observed across the other seven adverse outcomes examined.

**Research in context**

**Evidence before this study**

We searched for titles and abstracts of articles published in English in Medline and Embase from Jan 1, 1990, to Dec 1, 2018, and also searched references lists of identified studies. We constructed an initial search (search #1) to identify studies reporting on individuals discharged from psychiatric inpatient care: (discharge*) AND (mental* OR psychiatr* OR schiz* OR psychos* OR psychot* OR bipolar* OR manic* OR mania* OR personality disorder* OR psychopath* OR affective OR depress* OR neurotic* OR neurosis OR neuroses). We then did search #1 in combination with outcome-focused terms: (suicid* OR overdose* OR self-harm* OR self harm* OR self-injur* OR self injur* OR self-poison* OR self poison* OR parasuic*); (violen* OR aggress* OR assault* OR attack* OR homicid* OR kill* OR murder* OR reoffend* OR re-offend* OR recidiv* OR forensic); (death* OR mortal* OR died OR dying OR fatal* OR dead* OR lethal* OR loss of life); victim*; and accident*. In the literature, adverse outcomes have been examined predominantly as single outcomes in individual studies, with differences in underlying populations, settings, and outcome definitions and measures, rendering between-study comparison of risk estimates problematic. Although the evidence bases for suicide, non-fatal self-harm, and perpetration of violence post-discharge are substantial, reports on being a victim of violence are fewer, and almost no published studies have reported on risks of serious accidental harm or death in this population. We found no published studies that have investigated a broad range of adverse outcomes in a single large cohort. Therefore, comparison of absolute or relative risks for different types of fatal and non-fatal adverse outcomes post-discharge has not been possible.

**Added value of this study**

We did a national cohort study of almost 1·64 million Danish people, examining multiple fatal and non-fatal adverse outcomes among people discharged from their first inpatient psychiatric treatment episode, and calculated absolute and relative risks versus matched individuals of the same age and sex without a history of psychiatric admission, according to length of follow-up. As we investigated an array of serious adverse outcomes, direct comparison between multiple risks in a single cohort was possible. People discharged from psychiatric care were at higher risk of all eight adverse outcomes examined versus the comparator cohort. Around one in three people discharged from such care will have experienced at least one of the adverse outcomes examined at 10 years post-discharge, with absolute risks being highest in individuals diagnosed with a psychoactive substance use disorder at first discharge, and lowest in those diagnosed with a mood disorder. Risk of committing homicide was greater than that for becoming a homicide victim, although these absolute risks were minimal. Risks of suicide and self-harm were especially elevated within 3 months of first discharge, whereas risk elevations for accidental death, violent criminality, and being hospitalised due to violence were relatively homogeneous across the 10 year follow-up period.

**Implications of all the available evidence**

People discharged from psychiatric inpatient services are prone to experiencing various serious adverse outcomes. A multi-agency approach is thus needed to reduce these risks, and no single preventive measure will suffice. Some members of the general public and sections of the popular media often perceive and portray people with serious mental illnesses as being dangerous individuals. Although one in ten patients (and almost one in five men) will commit a violent crime within 10 years of their first discharge, only a small fraction of such individuals will commit homicide. Discharged patients are also at increased risk of dying by homicide and being hospitalised because of interpersonal violence, indicating a need to increase post-discharge liaison between multiple health-care and other public agencies to safeguard these individuals. Monitoring patients closely during the period of transition from inpatient to community care is crucial to minimise risk of self-harm and suicide. Long-term strategies are to indicated to ameliorate the risks of accidental death and interpersonal violence.

## Methods

### Study cohort

We did a national register-based cohort study, using the Danish Civil Registration System, which includes information on vital status and sex for all Danish residents born since 1968, to delineate the cohort.^17^ The personal identification numbering system in the Civil Registration System enabled complete linkage to data in the Psychiatric Central Research Register,^18^ the Register of Causes of Death,^19^ the National Patient Register,^20^ and the National Crime Register for the entire study period.^21^ Cohort members were individuals born in Denmark during 1967–2000, with two Danish-born parents, who were alive and still residing in the country at the time of their 15th birthday. We excluded people who had been discharged from inpatient psychiatric care before age 15 years, and individuals admitted to psychiatric emergency rooms without transfer to an inpatient ward. Every discharged patient was matched with 25 comparator cohort members on the basis of date of birth (±1 day) and sex. These comparators were living individuals who were residing in Denmark, and had not been admitted to inpatient psychiatric services on or before their index date (ie, the date of first discharge for the person to whom each comparator was matched). Some comparators, however, would have been admitted to a psychiatric unit after their index date, because not conditioning on future exposure information was necessary for delineating an unbiased cohort study.^22^ We followed the discharged cohort and the matched comparators from index date until the outcome of interest, death, emigration, or Dec 31, 2015, whichever came first. Thus, we censored the follow-up time of individuals who emigrated, but did not interval-censor subsequent periods during which cohort members were readmitted for further inpatient psychiatric care, admitted as inpatients to general hospitals, or imprisoned in correctional facilities.

The Danish Data Protection Agency approved this study and data access was granted by the Danish Health Data Authority and Statistics Denmark.

### Classification of outcomes and covariates

The Register of Causes of Death enabled identification of persons who died by any cause and specifically by suicide, homicide, and accident according to ICD-8^23^ and ICD-10^24^ (ICD-9 was never implemented in Denmark). Suicide was classified as ICD-8 E950-E959 or ICD-10 X60-X84, Y87.0; homicide as ICD-8 E960-E969 or ICD-10 X85-Y09, Y87.1; and accidental death as ICD-8 E800-E949 or ICD-10 V01-X59, Y85-Y86. We identified perpetrators of homicide and other violent crimes from the National Crime Register. Violent offences included homicide, assault, robbery, aggravated burglary or arson, possessing a weapon in a public place, violent threats, extortion, human trafficking, abduction, kidnapping, rioting and other public order offences, terrorism, and sexual offences. We examined the date of first crime committed after first discharge. For crimes for which this date was unknown (0·3%), the date of conviction was applied instead. The National Patient Register provided information on hospital admissions to treat injuries caused by interpersonal violence (reason-for-contact code 3, ICD-8 E960-E969, ICD-10 X85-Y09). Episodes of non-fatal self-harm were ascertained via the Psychiatric Central Research Register and the National Patient Register according to a previously developed classification algorithm.^25^ We obtained information on psychiatric admissions and diagnoses from the Psychiatric Central Research Register and applied the same ICD-8 and ICD-10 code ranges as reported by an earlier Danish registry study^26^ to generate the psychiatric diagnostic classification (appendix).

### Statistical analyses

We did Cox regression survival analyses to estimate risks in people discharged from psychiatric inpatient care relative to the comparator cohort as hazard ratios (HRs).^27^ To account for the matched design, we fitted stratified models according to the matched sets. Because the proportional hazards assumption did not hold for all outcomes examined, we calculated HRs separately for these follow-up periods: first 3 months; 3 months and 1 day to 6 months; 6 months and 1 day to 12 months; 1 year and 1 day to 5 years; 5 years and 1 day to 10 years; more than 10 years. Because homicide occurs so infrequently, we were unable to estimate HRs specific to the follow-up periods for homicide perpetration or victimisation. Right-censoring was applied at date of emigration from Denmark, date of death, or the final date of the observation period (Dec 31, 2015). We calculated all-person and sex-specific cumulative incidence percentages and their respective 95% CIs at 10 years of follow-up separately for the discharged patient cohort and for the matched comparators. In doing so, we accounted for competing risks because crude calculation of the cumulative incidence as the complement of the Kaplan-Meier estimate of overall survival overestimates the probability of the event of interest occurring when competing risks are present.^28^, ^29^

### Role of the funding source

The funders of the study had no role in the study design, data collection, data analysis, data interpretation, or writing of the report. The corresponding author had full access to all the data in the study, and had final responsibility for the decision to submit for publication.

## Results

The national cohort consisted of 62 922 people who had been discharged from inpatient psychiatric services and 1 573 050 who had never been a psychiatric inpatient. Table 1 shows the clinical and demographic characteristics of the discharged cohort. The most common diagnostic categories at first discharge were neurotic, stress-related, and somatoform disorders, mood disorders, and psychoactive substance use disorders. The median age at first discharge was 24 years (IQR 11), the median length of stay was 7 days (IQR 31), and most admissions were voluntary. 12 048 (19·1%) discharged patients had a previous history of self-harm before their first inpatient admission, 1743 (2·8%) had been hospitalised because of violence, and 5523 (8·8%) had a history of committing violent crimes.

**Table 1:** Sociodemographic and clinical characteristics of cohort members at first discharge from inpatient psychiatric care

	**Discharged cohort (n=62 922)**
**Sex**	
Male	30 805 (49·0%)
Female	32 117 (51·0%)
**Age at first discharge, years**	
15–19	16 222 (25·8%)
20–24	17 220 (27·4%)
25–29	11 725 (18·6%)
30–34	8366 (13·3%)
35–39	5572 (8·9%)
≥40	3817 (6·1%)
Psychiatric diagnostic category*	
Psychoactive substance misuse	13 913 (22·1%)
Schizophrenia and related disorders	10 119 (16·1%)
Mood disorders	16 114 (25·6%)
Neurotic, stress-related, and somatoform disorders	22 980 (36·5%)
Personality disorders	8954 (14·2%)
All other disorders combined	10 284 (16·3%)
**Type of admission**	
Voluntary	59 078 (93·9%)
Involuntary	3828 (6·1%)
Unknown	16 (<0·1%)
**Length of inpatient stay**	
Up to 7 days	31 840 (50·6%)
8–30 days	14 934 (23·7%)
31 days to 6 months	14 486 (23%)
More than 6 months	1662 (2·6%)
**History at first admission**	
Self-harm	12 048 (19·1%)
Hospitalised because of violence	1743 (2·8%)
Violent criminality	5523 (8·8%)

Data are n (%).

* These diagnostic categories are not mutually exclusive.

For all outcomes examined, HRs were increased among people who had been discharged from psychiatric inpatient care relative to those in the comparator cohort, with the largest HRs consistently observed during the first 3 months post-discharge (figure; appendix p 2). For all-cause mortality, suicide, and non-fatal self-harm, HRs were especially elevated during this initial post-discharge period, and decreased over time, but risk remained higher in the discharged cohort than in the matched comparison cohort in the long term. There was a steeper decline in the HRs for suicide and for non-fatal self-harm than for all-cause mortality over time. By contrast, although HR patterns for accidental death, violent criminality, and hospitalisation due to violence showed that risks were greatest within the first 3 months post-discharge, the attenuations in HR in the long term were smaller than those for suicide and self-harm. The ratios of HRs for adverse outcomes in the short term (≤3 months) versus the long term (>10 years) varied considerably across the outcomes examined (appendix): for suicide and non-fatal self-harm, the HRs within the first 3 months of discharge were more than ten times greater than those for the period beyond 10 years post-discharge, whereas the equivalent ratios were less than two for accidental death, violent criminality, and hospitalisation due to violence.

**Figure:**
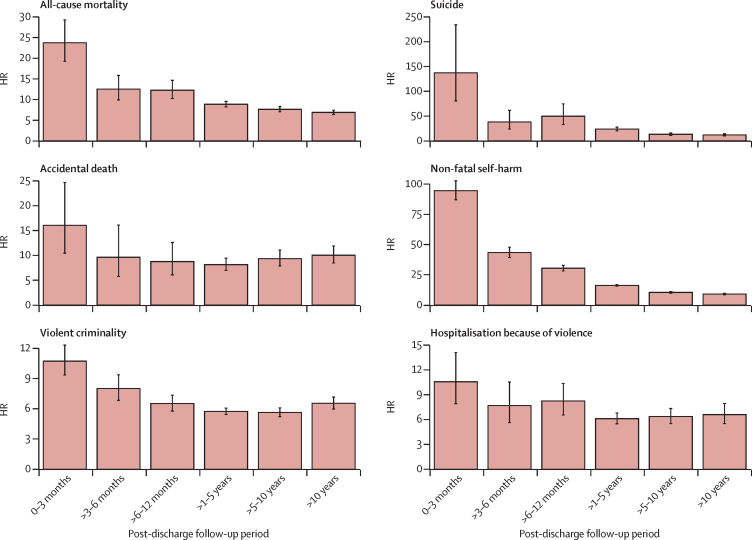
Adverse outcomes by post-discharge follow-up period Error bars show 95% CIs. A table of values is provided in the appendix (p 2). HR=hazard ratio.

In the cohort of patients discharged from inpatient psychiatric care, non-fatal self-harm had the highest incidence rates (19 136·8 per 100 000 person-years in the first 3 months) during all follow-up periods among all adverse outcomes assessed, whereas violent criminality had the highest incidence rates in the matched comparison cohort (281·6 per 100 000 person-years; appendix). Incidence rates decreased over time following discharge for all adverse outcomes. Within the first 10 years of follow-up, the cumulative incidence of each individual adverse outcome was higher among individuals discharged from inpatient psychiatric care than among those without a history of psychiatric admission, and the proportion of discharged individuals with at least one adverse outcome was 32·0% (95% CI 31·6–32·5), compared with 3·5% (3·5–3·6) of the matched comparators (table 2). Within 10 years of first discharge, the estimated cumulative incidence rates among discharged patients were 4·8% (4·6–5·0) for death from any cause, 1·5% (1·4–1·6) for suicide, 1·2% (1·1–1·3) for accidental death, 10·5% (10·2–10·8) for perpetration of a violent crime, 1·9% (1·8–2·0) for hospitalisation due to violence, and 22·5% (22·2–22·9) for self-harm. For the same period, the absolute risk of perpetrating homicide (0·17% [0·14–0·21]) was higher than the risk of being a victim of homicide (0·045% [0·028–0·071]) among those discharged.

**Table 2:** Absolute risk (cumulative incidence) of adverse outcomes at 10 years of follow-up

		**All**		**Men**		**Women**	
		n	Cumulative incidence,% (95% CI)	n	Cumulative incidence,% (95% CI)	n	Cumulative incidence,% (95% CI)
Discharged cohort		N=62 922	..	N=30 805	..	N=32 117	..
	All-cause mortality	2110	4·8 (4·6–5·0)	1519	7·0 (6·7–7·4)	591	2·6 (2·4–2·8)
	Suicide	691	1·5 (1·4–1·6)	493	2·2 (2·0–2·4)	198	0·84 (0·73–0·97)
	Accidental death	502	1·2 (1·1–1·3)	392	1·9 (1·7–2·1)	110	0·49 (0·41–0·60)
	Homicide victim	19	0·045 (0·028–0·071)	14	0·070 (0·040–0·119)	5	0·021 (0·008–0·050)
	Homicide perpetrator	77	0·17 (0·14–0·21)	59	0·27 (0·21–0·35)	18	0·075 (0·046–0·120)
	Self-harm	11 694	22·5 (22·2–22·9)	5230	21·1 (20·6–21·7)	6464	23·9 (23·4–24·4)
	Violent criminality	5045	10·5 (10·2–10·8)	4198	17·9 (17·4–18·4)	847	3·5 (3·3–3·8)
	Hospitalised because of violence	880	1·9 (1·8–2·0)	620	2·7 (2·5–2·9)	260	1·1 (1·0–1·3)
	Died, self-harmed, committed violent crime, or was hospitalised because of violence	16 409	32·0 (31·6–32·5)	9107	37·1 (36·5–37·8)	7302	27·2 (26·7–27·8)
Matched comparator cohort		N=1 573 050	..	N=770 125	..	N=802 925	..
	All-cause mortality	5896	0·57 (0·55–0·58)	4089	0·80 (0·78–0·83)	1807	0·34 (0·33–0·36)
	Suicide	763	0·075 (0·070–0·081)	638	0·13 (0·12–0·14)	125	0·024 (0·020–0·029)
	Accidental death	1428	0·13 (0·13–0·14)	1175	0·22 (0·21–0·24)	253	0·046 (0·041–0·052)
	Homicide victim	96	0·009 (0·007–0·011)	58	0·011 (0·008–0·014)	38	0·007 (0·005–0·009)
	Homicide perpetrator	106	0·010 (0·008–0·012)	96	0·019 (0·015–0·023)	10	0·002 (0·001–0·004)
	Self-harm	15 650	1·4 (1·4–1·4)	7178	1·3 (1·3–1·4)	8472	1·5 (1·4–1·5)
	Violent criminality	18 674	1·6 (1·6–1·6)	16 860	3·0 (2·9–3·0)	1814	0·32 (0·30–0·33)
	Hospitalised because of violence	3372	0·30 (0·29–0·31)	2867	0·52 (0·50–0·54)	505	0·091 (0·083–0·099)
	Died, self-harmed, committed violent crime, or was hospitalised because of violence	40 030	3·5 (3·5–3·6)	28 039	5·0 (5·0–5·1)	11 991	2·1 (2·1–2·1)

Men discharged from inpatient psychiatric care were more likely to have at least one of the adverse outcomes examined at 10 years post-discharge than were discharged women (table 2). Discharged men had higher absolute risks at 10 years of follow-up for all outcomes except non-fatal self harm (cumulative incidence 21·1% [20·6–21·7] among men *vs* 23·9% [23·4–24·4] among women). Of men discharged from psychiatric care, 7·0% (6·7–7·4) will have died by any cause and 17·9% (17·4–18·4) will have committed a violent crime within 10 years of first discharge. The absolute risks of perpetrating and being a victim of homicide were slightly higher in men than in women.

Absolute risks of at least one of the adverse outcomes occurring within 10 years of first discharge were also estimated by psychiatric diagnostic category (table 3). 49·4% of people diagnosed with a psychoactive substance use disorder at first discharge will have died by any cause, presented to hospital after harming themselves, committed a violent crime, or been hospitalised because of violence within 10 years, compared with 24·4% of those diagnosed with a mood disorder. Post-discharge absolute risk for all-cause mortality within 10 years was also highest among people diagnosed with a psychoactive substance use disorder (10·2% [9·5–10·8]), and was lowest among those with a mood disorder diagnosis (3·1% [2·8–3·5]).

**Table 3:** Absolute risk (cumulative incidence) of any of the examined adverse outcomes occurring within 10 years of first discharge by psychiatric diagnostic category

	**n**	Cumulative incidence, % (95% CI)*
**All discharged individuals (n=62 922)**		
Psychoactive substance misuse	5543	49·4 (48·4–50·4)
Schizophrenia and related disorders	2464	29·5 (28·5–30·5)
Mood disorders	3165	24·4 (23·6–25·2)
Neurotic, stress-related, and somatoform disorders	5429	29·8 (29·0–30·5)
Personality disorders	2963	38·0 (36·9–39·1)
All other disorders combined	2655	32·3 (31·2–33·4)
**Discharged men (n=30 805)**		
Psychoactive substance misuse	4294	51·5 (50·3–52·6)
Schizophrenia and related disorders	1531	30·9 (29·5–32·2)
Mood disorders	1277	27·0 (25·6–28·4)
Neurotic, stress-related, and somatoform disorders	2707	34·5 (33·4–35·7)
Personality disorders	1250	43·6 (41·7–45·5)
All other disorders combined	1295	37·1 (35·3–38·8)
**Discharged women (n=32 117)**		
Psychoactive substance misuse	1249	43·1 (41·1–45·0)
Schizophrenia and related disorders	933	27·3 (25·8–28·9)
Mood disorders	1888	22·9 (21·9–23·8)
Neurotic, stress-related, and somatoform disorders	2722	26·1 (25·2–27·0)
Personality disorders	1713	34·5 (33·1–35·8)
All other disorders combined	1360	28·8 (27·5–30·2)

* Indicates absolute risk of dying (by any cause), presenting to hospital following self-harm, committing a violent crime, or being hospitalised due to violence within 10 years of first discharge.

## Discussion

In this epidemiological study, we investigated a comprehensive array of fatal and non-fatal adverse outcomes among people discharged from their first inpatient psychiatric stay in a single national cohort. As hypothesised, these individuals were at increased risk for all adverse outcomes examined compared with people without a history of inpatient psychiatric admission, with almost one in three discharged individuals having at least one of the examined outcomes within 10 years of their first discharge. The absolute risks of having at least one of the array of adverse outcomes within this timeframe were highest in people diagnosed with a psychoactive substance use disorder at first discharge, and lowest in those diagnosed with a mood disorder. Relative risks (HRs) were consistently highest within the first 3 months post-discharge, especially for suicide (137 times higher than the matched comparators) and non-fatal self-harm (100 times higher), whereas accidental death, violent criminality, and hospitalisation due to violence showed less elevated risks and less variability across the whole follow-up period. Within 10 years after discharge, almost one in four discharged patients will have been treated in hospital following self-harm, and almost one in five discharged men will have committed a violent crime. Individuals who had been discharged were at higher risk of perpetrating homicide than becoming a victim, although these two outcomes were extremely rare. Comparing sex-specific absolute risks among people discharged from psychiatric care, men had higher cumulative incidence values than women for all outcomes except for non-fatal self-harm, for which women were at higher risk.

Previous studies reporting on post-discharge risks have tended to examine and report on single outcomes, focusing usually on either risk of harm to self or harm to others. This study is unique in its consideration of an array of eight serious fatal and non-fatal adverse outcomes in this especially at-risk population. Studies examining suicide are especially numerous in the literature.^5^ The results of our study are consistent with those reported previously. Our time-to-event analysis confirmed that risk, especially for suicide, is highest within 3 months of discharge,^3^, ^5^ and non-fatal self-harm showed a similar pattern. A reason for this heightened suicidality risk during the period directly following discharge might be an incomplete recovery and sudden withdrawal from inpatient care,^3^ leading to a distressing transition back into the community. Access to multiple means of harming oneself at this time could further exacerbate risk.

Previous studies have shown elevated risks of violent crime victimisation,^6^, ^7^, ^10^ and of dying by homicide specifically,^8^ among psychiatric patients than among the remainder of the population. One possible explanation for this finding is that excessive comorbid misuse of alcohol or illicit drugs disinhibit people to such a degree that they place themselves in dangerous situations or lose their capacity to identify and react appropriately to potential threats.^7^ Hazardous social environments and peer networks are also likely to contribute to the elevated risk.^9^ Other studies have shown that psychiatric patients are at higher risk of perpetrating violent crimes, including homicides.^11^, ^12^, ^13^, ^14^, ^15^ The presence of comorbid substance misuse markedly increases the risk of a discharged patient being violent,^11^ but further research is needed to elucidate the causal pathway. Previous research has shown that suicide, violent crime perpetration, and premature mortality have shared modifiable risk factors, such as substance misuse and a history of self-harm and criminality, which could be a focus of future interventions.^30^

Only two previously published studies have examined both crime perpetration and victimisation in single cohorts of individuals with mental illnesses.^31^, ^32^ From their investigation of 172 people diagnosed with schizophrenia or schizoaffective disorder in Los Angeles, USA, in 1989–91, Brekke and colleagues^31^ reported that cohort members were considerably more likely to become violent crime victims than to be arrested for perpetrating violence. By contrast, using interlinked public health and criminal justice data sources, Pandiani and colleagues^32^ reported virtually equivalent risks of perpetration (6·6%) and victimisation (7·1%) in their cohort of 2610 individuals diagnosed with serious mental illnesses in 13 rural counties of Vermont State, USA.^32^ Our national register-based cohort study of almost 63 000 people discharged from inpatient psychiatric care indicates higher risk for perpetrating than for becoming a victim of homicide in this population, albeit with very low absolute risks for both outcomes. Furthermore, even if the incidence of homicide perpetration among people with serious mental illnesses could somehow be reduced to the background rate among people without such illnesses, the majority of homicide cases in the population would still occur. The relationship between severe mental illness and elevated risk of serious violent crime is complex; for instance, a study conducted in the UK reported that when people with mental illness became victims of homicide, the perpetrator often also had a psychiatric disorder.^33^

Accidental death among people with mental illnesses has been examined considerably less frequently than have suicide, self-harm, and violent crime.^1^, ^34^ Our study indicated that risk of dying accidentally was less elevated than were risks of suicide and non-fatal self-harm. Nonetheless, risk of accidental death was markedly increased versus that of people without history of inpatient psychiatric admission, and the cumulative incidence estimates indicate that around one in 50 male former inpatients and one in 200 female former inpatients die accidentally within 10 years of their first discharge.

This population-based study has examined a comprehensive set of fatal and non-fatal adverse outcomes in one large cohort of people discharged from inpatient psychiatric services. This innovative approach enabled direct comparison of absolute and relative risks with additional fine-grained analysis according to follow-up period, sex, and psychiatric diagnostic category at first discharge. The large cohort, based on administrative data of high quality^17^ and unique to the Nordic countries, provided sufficient statistical power and precision for examining even the rarest external cause of death: homicide. The comparison of perpetration and victimisation data in the same study cohort is an especially novel feature.

However, our study had several limitations. First, the high absolute risks calculated will have been underestimated because we could not ascertain some specific types of adverse outcome with the registry data available. Thus, we did not capture serious, non-fatal accidents that resulted in long-term impairment or disability, self-harm episodes occurring in the community without subsequent hospital presentation, or cases of interpersonal violence that did not result in a criminal conviction. As linked police-reported violent crime victimisation events were only available from 2001, we could not include this variable in the outcome array, although we did examine hospitalisation due to violence as a proxy for victimisation. Second, since the study was done using data collected for administrative rather than research purposes, the availability of covariates was limited; for instance, no detailed clinical information was available regarding treatment received. Third, the numbers of homicide perpetrators and victims were too low to estimate relative risks specific to varying periods of follow-up, unlike with the other six outcomes examined. Finally, we used interlinked registry data from Denmark, a nation with low incidence of homicide^35^ and suicide.^36^ The 3-year average homicide incidence for 2014–2016 was 1·08 per 100 000 population in Denmark, which was somewhat higher than the rates in Spain (0·66 per 100 000 population), Italy (0·75 per 100 000 population), Germany (0·96 per 100 000 population), Australia (0·99 per 100 000 population), and the UK (1·03 per 100 000 population), somewhat lower than the rates in France (1·39 per 100 000 population) and Canada (1·61 per 100 000 population), and considerably lower than the rate in the USA (4·93 per 100 000 population).^35^ In 2016, the range in national suicide incidence among these countries was narrower than the range in homicide incidence, with Denmark having 9·2 suicides per 100 000 population compared with 5·5 per 100 000 population in Italy and 13·7 per 100 000 population in the USA.^36^ Therefore, the magnitude of the observed absolute risk estimates in Denmark might differ from those that would be seen in other countries. However, there is no reason to believe that the overall risk patterns would be substantially different from those in most other western countries, although this assumption cannot be tested empirically because of the dearth of interlinked national registers beyond Scandinavia.

This comprehensive national study has quantified the degree to which people discharged from inpatient psychiatric services are at elevated risk across an array of fatal and non-fatal adverse outcomes. The significant increase in the cumulative incidence of all-cause mortality—especially for men, with one in 14 dying within 10 years after first discharge—underlines the degree to which these individuals are at risk. Reducing risks across so many outcome domains cannot be achieved by a single preventive measure, but will require well-coordinated strategies involving enhanced levels of cooperation between public agencies. Psychiatric services must address the multiple susceptibilities that discharged patients face, ensure adequate assessment and management of these diverse risks, and safeguard these at-risk individuals and the communities that they are discharged back into. Reduction of the risks of self-harm and suicide will require more intensive prevention during the initial post-discharge period, including care planning on discharge and proactive early follow-up. Such steps are crucial, considering the elevated risk immediately after discharge when patients are in close proximity to care. Other risks, such as violent criminality, homicide perpetration and victimisation, and accidental death, are more likely to need longer-term aftercare, social support, and interventions for substance misuse, in addition to short-term preventive measures. Future research should focus on elucidating causal pathways and developing evidence for effective preventive strategies that target the assortment of adverse outcomes that occur much more commonly during and following the potentially hazardous transition from inpatient to community care.
