# Supplementary material for: Multiple adverse outcomes following first discharge from inpatient psychiatric care: a national cohort study
Source: Lancet Psychiatry. 2019 Jul;6(7):582–9. doi: 10.1016/S2215-0366(19)30180-4 (PMC6586943; doi:10.1016/S2215-0366(19)30180-4)
Supplement: Supplementary appendix [file mmc1.pdf]

THE LANCET
Psychiatry

Supplementary appendix

This appendix formed part of the original submission and has been peer reviewed. We post it as supplied by the authors.

Supplement to: Walter F, Carr MJ, Mok PLH, et al. Multiple adverse outcomes following first discharge from inpatient psychiatric care: a national cohort study. *Lancet Psychiatry* 2019; published online June 3. http://dx.doi.org/10.1016/S2215-0366(19)30180-4.

eTable 1: Classification of psychiatric diagnostic categories at first inpatient episode according to ICD-10 and equivalent ICD-8 codes

Diagnostic category	ICD-10 codes	ICD-8 codes
Psychoactive substance abuse	F10-F19	291.x9, 294.39, 303.x9, 303.20, 303.28, 303.90, 304.x9
Schizophrenia and related disorders	F20-F29	295.x9, 296.89, 297.x9, 298.29-298.99, 299.04, 299.05, 299.09, 301.83
Mood disorders	F30-F39	296.x9 (excluding 296.89), 298.09, 298.19, 300.49, 301.19
Neurotic, stress-related, and somatoform disorders	F40-F48	300.x9 (excluding 300.49), 305.x9, 305.68, 307.99
Personality disorders	F60	301.x9 (excluding 301.19), 301.80, 301.81, 301.82, 301.84
All other disorders combined	F00-F09<br>F50<br>F70-F79<br>F84<br>F90-F98	290.09, 290.10, 290.11, 290.18, 290.19,<br>292.x9, 293.x9, 294.x9, 309.x9<br>305.60, 306.50, 306.58, 306.59,<br>311.xx, 312.xx, 313.xx, 314.xx, 315.xx<br>299.00, 299.01, 299.02, 299.03<br>306.x9, 308.0x

eTable 2: HRs specific to varying follow-up periods among discharged persons and the matched comparison cohort

Adverse outcomes	First 3 months:		>3, ≤6 months:		>6, ≤12 months:		>1, ≤5 years:		>5, ≤10 years:		10 years and more:	
	**HR**	**(95% CI)**	**HR**	**(95% CI)**	**HR**	**(95% CI)**	**HR**	**(95% CI)**	**HR**	**(95% CI)**	**HR**	**(95% CI)**
All-cause mortality	23.7	(19.2, 29.3)	12.5	(9.9, 15.8)	12.3	(10.3, 14.7)	8.9	(8.2, 9.6)	7.6	(7.0, 8.3)	6.9	(6.4, 7.4)
Suicide	137.4	(80.7, 234.1)	38.3	(23.8, 61.6)	49.8	(33.2, 74.7)	23.8	(20.4, 27.8)	13.3	(11.1, 16.0)	12.2	(10.3, 14.5)
Accidental death	16.0	(10.4, 24.7)	9.6	(5.7, 16.1)	8.7	(6.1, 12.6)	8.1	(7.0, 9.4)	9.3	(7.9, 11.1)	10.0	(8.5, 11.9)
Self-harm	94.5	(87.1, 102.7)	43.3	(39.3, 47.7)	30.3	(28.2, 32.7)	16.4	(15.7, 17.0)	10.6	(10.0, 11.2)	9.2	(8.5, 9.9)
Violent criminality	10.7	(9.4, 12.3)	8.0	(6.8, 9.4)	6.5	(5.8, 7.4)	5.7	(5.4, 6.1)	5.6	(5.2, 6.1)	6.5	(6.0, 7.2)
Hospitalised due to violence	10.6	(7.9, 14.1)	7.7	(5.6, 10.5)	8.2	(6.6, 10.4)	6.1	(5.5, 6.8)	6.4	(5.5, 7.3)	6.6	(5.5, 7.9)

Ratios of HR for 10 years or more vs. HR for first 3 months:
All-cause mortality (ratio 3.4; 95% CI 2.8, 4.3); Suicide (ratio 11.2; 95% CI 6.4, 19.7); Accidental death (ratio 1.6; 95% CI 1.0, 2.5); Self-harm (ratio 10.3; 95% CI 9.2, 11.5); Violent criminality (ratio 1.6; 95% CI 1.4, 1.8); Hospitalised due to violence (ratio 1.6; 95% CI 1.1, 2.3)

eTable 3: Incidence rates per 100,000 person-years specific to varying follow-up periods among discharged persons and the matched comparison cohort

**Adverse outcomes**	**First 3 months:**		**>3, ≤6 months:**		**>6, ≤12 months:**		**>1, ≤5 years:**		**>5, ≤10 years:**		**10 years and more:**	
	n	**Rate**	***n***	**Rate**	***n***	**Rate**	***n***	**Rate**	***n***	**Rate**	***n***	**Rate**
**Persons discharged** *(N=62,922)*												
All-cause mortality	170	1091.5	106	693.5	180	605.9	917	463.2	737	459.9	928	590.3
Suicide	88	565.0	43	281.3	70	235.6	311	157.1	179	111.7	205	130.4
Accidental death	34	218.3	20	130.8	39	131.3	225	113.7	184	114.8	194	123.4
Self-harm	2892	19,136.8	1174	8137.5	1598	5839.5	4430	2607.9	1599	1257.0	1020	866.2
Violent criminality	467	3009.9	378	2500.1	612	2099.9	2481	1320.5	1107	760.3	752	557.0
Hospitalised due to violence	67	430.5	51	334.1	97	327.4	425	216.5	240	152.7	149	98.4
**Matched comparison cohort** *(N=1,573,050)*												
All-cause mortality	179	45.9	212	55.4	369	49.5	2613	52.4	2523	61.8	3647	88.2
Suicide	16	4.1	28	7.3	35	4.7	330	6.6	354	8.7	458	11.1
Accidental death	53	13.6	52	13.6	113	15.2	698	14.0	512	12.5	526	12.7
Self-harm	751	192.7	704	183.9	1408	189.1	8006	161.3	4780	118.5	3742	92.2
Violent criminality	1097	281.6	1047	273.7	1914	257.3	9717	196.2	4899	121.8	3231	80.0
Hospitalised due to violence	156	40.0	169	44.1	297	39.9	1768	35.5	982	24.1	605	14.7
